# Antarctic Glacial Meltwater Impacts the Diversity of Fungal Parasites Associated With Benthic Diatoms in Shallow Coastal Zones

**DOI:** 10.3389/fmicb.2022.805694

**Published:** 2022-03-04

**Authors:** Doris Ilicic, Jason Woodhouse, Ulf Karsten, Jonas Zimmermann, Thomas Wichard, Maria Liliana Quartino, Gabriela Laura Campana, Alexandra Livenets, Silke Van den Wyngaert, Hans-Peter Grossart

**Affiliations:** ^1^Department of Experimental Limnology, Leibniz Institute of Freshwater Ecology and Inland Fisheries, Neuglobsow, Germany; ^2^Institute of Biological Sciences, Applied Ecology and Phycology, University of Rostock, Rostock, Germany; ^3^Botanic Garden and Botanical Museum Berlin-Dahlem, Freie Universität Berlin, Berlin, Germany; ^4^Institute for Inorganic and Analytical Chemistry, Friedrich-Schiller-University Jena, Jena, Germany; ^5^Department of Coastal Biology, Argentinean Antarctic Institute, Buenos Aires, Argentina; ^6^Department of Basic Sciences, National University of Luján, Luján, Buenos Aires, Argentina; ^7^Department of Biology, University of Turku, Turku, Finland; ^8^Institute of Biochemistry and Biology, University of Potsdam, Potsdam, Germany

**Keywords:** Antarctica, aquatic fungi, Chytridiomycota, phytoplankton host, salinity gradient, Illumina amplicon sequencing, Carlini Station

## Abstract

Aquatic ecosystems are frequently overlooked as fungal habitats, although there is increasing evidence that their diversity and ecological importance are greater than previously considered. Aquatic fungi are critical and abundant components of nutrient cycling and food web dynamics, e.g., exerting top-down control on phytoplankton communities and forming symbioses with many marine microorganisms. However, their relevance for microphytobenthic communities is almost unexplored. In the light of global warming, polar regions face extreme changes in abiotic factors with a severe impact on biodiversity and ecosystem functioning. Therefore, this study aimed to describe, for the first time, fungal diversity in Antarctic benthic habitats along the salinity gradient and to determine the co-occurrence of fungal parasites with their algal hosts, which were dominated by benthic diatoms. Our results reveal that Ascomycota and Chytridiomycota are the most abundant fungal taxa in these habitats. We show that also in Antarctic waters, salinity has a major impact on shaping not just fungal but rather the whole eukaryotic community composition, with a diversity of aquatic fungi increasing as salinity decreases. Moreover, we determined correlations between putative fungal parasites and potential benthic diatom hosts, highlighting the need for further systematic analysis of fungal diversity along with studies on taxonomy and ecological roles of Chytridiomycota.

## Introduction

Fungi are morphologically, phylogenetically, and functionally diverse. They constitute a well-founded component of terrestrial ecology due to more than 100 years of research that has highlighted their role in biogeochemical cycling and promoting biodiversity (Peay et al., 2016). Aquatic ecosystems, in particular the oceans, are frequently overlooked as fungal habitats with a systematic analysis of fungal diversity and their ecological roles still in their infancy. However, there is increasing evidence that fungal diversity in aquatic ecosystems is greater and more important than previously considered (Shearer et al., 2007; Amend et al., 2019; Grossart et al., 2019). Despite sampling efforts of fungi in aquatic habitats being low compared to terrestrial environments (Rojas-Jimenez et al., 2017), molecular analyses of environmental DNA samples reveal a great diversity of novel fungal sequences, the so-called Dark Matter Fungi (Grossart et al., 2016). Although recent advances in DNA-sequencing technology have revealed that fungi are highly abundant in marine environments (Comeau et al., 2016; Taylor and Cunliffe, 2016; Tisthammer et al., 2016; Hassett et al., 2019a; Banos et al., 2020), their ecological functions and interactions with other microorganisms remain largely unexplored and missing from current general concepts (Amend et al., 2019; Richards et al., 2021).

Based on recent studies, marine fungal communities are dominated by members of the phylum Ascomycota (Tisthammer et al., 2016; Amend et al., 2019; Hassett et al., 2019a) and Chytridiomycota (Comeau et al., 2016; Hassett and Gradinger, 2016; Hassett et al., 2017; Richards et al., 2021). Chytridiomycota, frequently referred to as chytrids, are often recognized as zoosporic virulent parasites on phytoplankton which play significant roles in controlling population sizes of their hosts (Ibelings et al., 2004; Kagami et al., 2004; Rasconi et al., 2011) and altering food web structure by transferring carbon and energy between trophic levels (Rasconi et al., 2012, 2020; Sime-Ngando and Twiss, 2012; Klawonn et al., 2021b). The majority of quantitative studies of zoosporic fungi to date have been laboratory-based, with only a small number of field studies conducted to assess their importance into the broader environment. In addition, zoosporic fungi are difficult to identify using solely morphological characteristics. Although direct examination of the diversity of zoosporic fungi using high-throughput sequencing has been increasingly applied (Monchy et al., 2011; Tedersoo et al., 2015, 2018; Seto et al., 2017; Li et al., 2021; Richards et al., 2021), data are still lacking for polar regions.

Microphytobenthos (MPB) comprise phototrophic benthic microalgae living in intertidal and shallow subtidal sediments and hard substrates, among which benthic diatoms are typically the dominant organisms and are known as preferential hosts for fungal parasites such as chytrids (Scholz et al., 2016a). Although the amount of research on fungal parasites infecting pelagic forms is growing, data about microphytobenthic taxa are sparse (Wulff et al., 2009; Scholz et al., 2016b). Generally, benthic microalgae are among the main contributors to primary production in coastal zones, particularly those poor in nutrients and organic matter. They can contribute up to 42% of the marine benthic primary production (Woelfel et al., 2010). Specifically, according to Cahoon (1999), the global production of benthic microalgae ranges from 8.9 to 14.4 Gt C m^–2^ year^–1^, representing approximately 20% of the global ocean production. Hence, they are important suppliers of organic carbon to grazers and sediment feeding macro- and meiofauna (Middelburg et al., 2000; Oakes et al., 2010). Except for sediments, benthic microalgae are also a major and ecologically important components of epilithic biofilms (Cahoon, 1999). They exert further important ecological roles in marine shallow water environments as they occur at the sediment-water interface and thus directly influence, for example, vertical nutrient exchange processes (Sundbäck et al., 2000). Dissolved organic carbon is regularly released by benthic diatom excretion of extrapolymeric substances (EPS), which act as stabilizing compounds against hydrodynamic sediment erosion as well as bacterial substrate (de Brouwer et al., 2005; Aslam et al., 2012).

Marine environments, especially polar regions, which have historically experienced minimum human disturbance, are a vast reservoir of microbial diversity. Their clean air, water and ice are of great importance to science for understanding different aspects of the Earth’s environment. Hence, they have been drawing more attention and as such, they have not escaped the negative impacts of human activity. Their unique marine ecosystems are affected on local and regional scales by overfishing, pollution, introduction of invasive species and exploitation of mineral reserves, oil and gas (Aronson et al., 2011). Recently, in the light of global warming, the specific focus is on the loss of sea ice (Stammerjohn et al., 2008). During summer and winter, Antarctic sea ice does not show a significant overall trend (Parkinson and DiGirolamo, 2021). Nevertheless, south and west of the Antarctic Peninsula, one region has shown a persistent decline (Grosfeld et al., 2013). In February 2020 weather stations recorded the hottest temperature on record for Antarctica, reaching 18.3°C (64.9°F) (Rocha Francelino et al., 2021). This heatwave was the third major melt event of the 2019–2020 summer, following warm spells in November 2019 and January 2020. Consequently, polar aquatic ecosystems face local changes that include higher water temperatures and altered patterns in light penetration, dust deposition, sediment load and changes in salinity due to intensified glacier melt and subsequent terrestrial runoff (Eraso and Dominguez, 2007; Hernández et al., 2019). Similar climate-change-driven changes in abiotic factors were shown to affect microbial community structure, e.g., chytrid community composition in Arctic waters (Kilias et al., 2020), but for Antarctica, such data are scarce. Slow-moving or sedentary benthic communities in shallow nearshore areas are most vulnerable to these processes, with their species richness and diversity strongly impacted. Moon et al. (2015) discuss the impact of glacial retreat on epibenthic megafaunal assemblages on the West Antarctic Peninsula (WAP) and note significant differences in assemblages related to distance from the glacier, substrate grain size, and organic content. Furthermore, Braeckman et al. (2021) showed that benthic communities along the WAP shift from net autotrophy to net heterotrophy when affected by glacial meltwater. Nevertheless, more information is needed to gain a comprehensive understanding of the effects of glacial meltwater on benthic, especially fungal, communities. Although a growing amount of research focuses on fungal parasites in aquatic ecosystems, polar regions remain mainly unexplored. Rojas-Jimenez et al. (2017) reported early diverging lineages within Chytridiomycota and Cryptomycota as dominant among fungal sequences in the Antarctic Dry Valley lakes. Yet, in Antarctic marine systems, further research needs to be done to gain more detailed insights into their diversity and abundance changes and their ecological role under current climate change.

Despite the potentially important effect of fungal parasites on benthic diatoms, to our knowledge, no published experimental or field studies have been conducted on their diversity in Antarctic coastal waters. Therefore, in this study, we aim to qualitatively describe fungal community composition in Antarctic benthic habitats. Our main focus being on the diversity of parasitic fungi and the co-occurrence with their benthic microalgal hosts along the salinity gradient in Potter Cove, Antarctic Peninsula, which is impacted by a summer induced glacial meltwater runoff. We hypothesized that members of the fungal phylum Chytridiomycota predominate fungal taxa in Antarctic shallow coastal waters and exhibit a still unexplored biodiversity with many undescribed species.

## Materials and Methods

### Sites and Sample Collection

**FIGURE 1:**
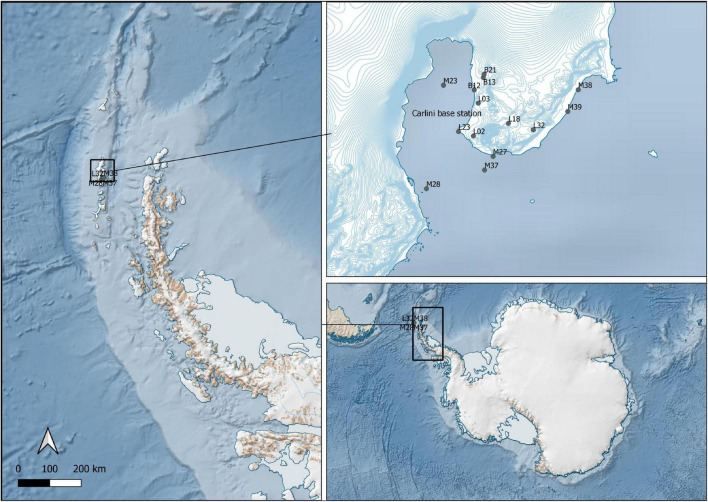
Sampling sites in the Potter Cove area (South Shetland Islands, western Antarctic Peninsula).

The study was conducted at the German-Argentine Dallmann Laboratory in the Argentine Scientific Station “Dr. Carlini,” which is located at Potter Cove, King George Island/25 de Mayo I, Antarctic Peninsula (62°14′S, 58°31′W). This area combines zones of glacier fronts, rocky shores and extensive seabed overlaid with sand or sediment. Sampling took place in January–February 2020 in several locations around Carlini Station and the Antarctic Specially Protected Area (ASPA) 132 (Figure 1). The sites included marine shallow water locations and open water locations down to 60 m depth, limnic locations that included lakes and ponds, and brackish meltwater runoff (Table 1). The area provided diverse sediment and biofilm habitats for the sampling of benthic diatoms. Sediment surface samples were taken using Plexiglas sediment corer, and biofilm samples were taken by scratching off the surface of at least five stones with a sterilized knife. Sampling of the ocean bottom was done with the help of Argentinian army divers. Each sample was divided into two parts and prepared the following way: (i) the part for DNA analysis was fixed with 70% ethanol, and the samples were kept at –20°C; (ii) the part for microscopy was fixed with Lugol’s solution.

**TABLE 1:** Sample points characteristics.

Sample	Location	Latitude	Longitude	Habitat	Sample type	Depth	Habitat specification
B21	Punta Elefante	62°14′ 08.0″ S	58° 38′ 54.5″ W	Brackish	Biofilm	0	Meltwater
B12	Casa de bomba	62°14′ 07.78″ S	58° 39′ 27.91″ W	Brackish	Sediment	0	Meltwater
B13	Casa de bomba	62°14′ 08.76″ S	58° 39′ 30.18″ W	Brackish	Sediment	0	Meltwater
L03	Peñón I	62°14′ 16.39″ S	58° 39′ 44.4″ W	Limnic	Biofilm	0	Pond
L18	Punta Stranger	62°14′ 46.77″ S	58° 39′ 47.05″ W	Limnic	Biofilm	0	Lake
L23	Punta Elefante	62°14′ 14.6″ S	58° 40′ 46.45″ W	Limnic	Biofilm	0	Lake
L02	Peñón I	62°14′ 27.15″ S	58° 40′ 39.23″ W	Limnic	Water	0	Pond
L32	Refugio Albatros	62° 15′ 07.44″ S	58° 39′ 33.89″ W	Limnic	Water	0	Meltwater
M26	Peñón I	62°14′ 50.22″ S	58° 40′ 52.18″ W	Marine	Biofilm	0	Shore
M27	Peñón I	62°14′ 50.22″ S	58° 40′ 52.18″ W	Marine	Biofilm	2	Ocean bottom
M28	Peñón de Pesca	62°14′ 16.5″ S	58° 42′ 44.2″ W	Marine	Biofilm	5	Ocean bottom
M37	Peñón I	62°14′ 50.01″ S	58° 40′ 81.13″ W	Marine	Biofilm	0	Shore
M38	Refugio Elefante	62°15′ 22.16″ S	58° 37′ 50.1″ W	Marine	Biofilm	0	Shore
M39	Refugio Elefante	62°15′ 24.16″ S	58° 38′ 33.31″ W	Marine	Biofilm	0	Shore
M14	A4	62°13′ 43.61″ S	58° 39′ 49.36″ W	Marine	Sediment	15	Ocean bottom
M15	A4	62°13′ 43.61″ S	58° 39′ 49.36″ W	Marine	Sediment	15	Ocean bottom
M23	A4	62°13′ 43.61″ S	58° 39′ 49.36″ W	Marine	Sediment	20	Ocean bottom

### DNA Extraction and Sequencing

DNA from the microorganisms in ethanol fixed sediment and biofilm samples was extracted according to a modified protocol described by Nercessian et al. (2005). Briefly, cell lysis was achieved using small (0.1–1 mm) zirconia-silica beads that were suspended in cetyltrimethyl ammoniumbromide (CTAB), to which anion surfactants sodium dodecyl sulfate and N-Lauroylsarcosin, proteinase K and phenol–chloroform–isoamylalcohol were added. DNA purification was facilitated by the addition of chloroform–isoamylalcohol and polyethylene glycol (PEG). Finally, DNA was precipitated at 4°C, washed with ethanol, air-dried and dissolved in ultra-pure water. The detailed protocol is available in Supplementary Material. PCR, library preparation and sequencing was undertaken at mrDNA laboratories (Shallow Waters, Texas, United States). The V8 region of the 18S rRNA gene was amplified using primers 18S-82F (5′- GAAACTGCGAATGGCTC-3′) and Ek-516R (5′- ACCAGACTTGCCCTCC-3′) (Hassett et al., 2019b) for molecular characterization of diatoms, followed by library preparation (2 × 300 bp) and sequencing on a MiSeq (Illumina) platform. This dataset will be referred to as EukSSU further in the text. For molecular characterization of fungi we amplified the LSU D1 region of rRNA using primers ITS4ngsF (5′-GCA TAT CAA TAA GCG SAG GAA-3′) and LF402R (5′-TTC CCT TTY ARC AAT TTC AC-3′) (Tedersoo et al., 2015). This dataset will be referred to as FunLSU further in the text.

### Taxonomic Identification and Amplicon Sequence Variant Generation

Sequences were processed in R (ver. 4.0.3) using the DADA2 pipeline (ver. 1.8) (Callahan et al., 2016). Primers were removed from demultiplexed reads using cutadapt ver. 3.5 (Martin, 2011). The primer-free sequences were then filtered and trimmed to remove low-quality sequences. The DADA2 algorithm was used to infer amplicon sequence variants (ASVs). Paired-end reads were merged to obtain full denoised sequences. Chimeric sequences were removed, with the exception of 18S rRNA chimeric sequences, which were identified and removed using DECIPHER online tool ver. 11.4 (Wright et al., 2012). 18S rRNA gene sequences were run against the SILVA SSU ver. 132 database (Quast et al., 2013) for identification of all eukaryotic taxa and LSU sequences were run against LSU database using RDP Classifier and Fungal LSU training set 11 (Wang et al., 2007) for identification of fungal taxa.

### Data Analysis

Data processing, visualizations and statistical analysis were performed in R. Prior to any analysis, singletons were removed from both EukSSU and FunLSU datasets. Differences in community structure in respect to water types between samples (beta diversity) were calculated using a Bray-Curtis dissimilarity measure, using *phyloseq* package (McMurdie and Holmes, 2013), and visualized through non-metric multidimensional scaling (NMDS) ordination. Permutational multivariate analysis of variance (PERMANOVA) was used to test the effect of different water types on community structure using the “Adonis” function in the R package *vegan* (Oksanen et al., 2019). The statistical significance was calculated with a *post hoc* pairwise *t*-test using “pairwise adonis” function (Martinez Arbizu, 2020) with 999 permutations. *P*-values of the pairwise *t*-test were adjusted with the Bonferroni method. To calculate alpha diversity values, *microbiome* package was used (Lahti and Shetty, 2017). The graphical representation of results was realized using the R package *ggplot2* (Wickham, 2016). Ternary plots were made using *ggtern* package (Hamilton and Ferry, 2018). Map of sampling points was generated in software QGIS 3.16.14 (QGIS Development Team, 2021) using a geospatial data package and visualization platform Quantarctica (Matsuoka et al., 2021).

### Network Analysis

To infer whether the presence of suitable hosts is correlated with the presence of fungal parasites in collected samples, we performed a co-occurrence network analysis using the SparCC algorithm (Friedman and Alm, 2012) implemented in FastSpar (Watts et al., 2019). Prior to the network analysis, ASVs with < 10% prevalence and occurring in less than three samples were removed from both EukSSU and FunLSU datasets. Data was further processed in R using *WGCNA* package (Langfelder and Horvath, 2008). We maintained the independence of both datasets and converted the correlation coefficients obtained from FastSpar into a topological overlap matrix (TOM) as the first step to identify modules of co-occurring taxa across our samples. Using parallel minimum (pmin) we calculated a consensus TOM from the EukSSU and FunLSU TOMs which was then used as an input for hierarchical average linkage clustering. To identify consensus modules we used cutreeDynamic with a “deepSplit” of two and “minModuleSize” of five. During this step ASVs are assigned to certain modules depending on their TOM-based topology. Further co-occurrence between identified EukSSU and FunLSU modules was determined by extracting their respective Eigen values and calculating Spearman rank correlation coefficients. Co-occurrence between individual EukSSU and FunLSU ASVs was calculated by multiplying for each ASV pair, the between module Spearman correlation, by the module membership (range 0–1) for each ASV. In practice, this ensures that the two ASVs with the highest module membership from each respective module are most strongly correlated, and corr values decrease with decreasing module membership. We visualized for selected module pairs, weighted correlation coefficients (> 0.5) between ASVs using Cytoscape 3.8.2 software.

### Microscopy

CalcoFluor White (CFW) staining approach along with epifluorescence microscopy, as suggested by Rasconi et al. (2009), was used to detect and identify fungal parasites attached to an algal hosts. CFW is a non-specific, fluorescent dye that binds to chitin in fungal cell walls but also cellulose, that is often present in cell wall of some algae and fungi-like organisms (Kagami et al., 2007; Priest et al., 2021). Pre-treatment of sediment samples was done using ultrasound (3 × 10 s) for the mechanical disruption of diatom-sand agglomerates (Scholz et al., 2014). Staining was performed as described in Klawonn et al. (2021a). Five microliter of CFW (1 mg/ml) was added to 1 ml sample and incubated for 15 min at room temperature. Samples were then transferred in Utermöhl counting chambers, after which they were incubated for 10 min, allowing the cells to sink to the bottom of the chamber. For further visualization, Nikon Eclipse Ti2 inverted microscope was used and the samples were screened for fungal parasites on 600x magnification.

## Results

**FIGURE 2:**
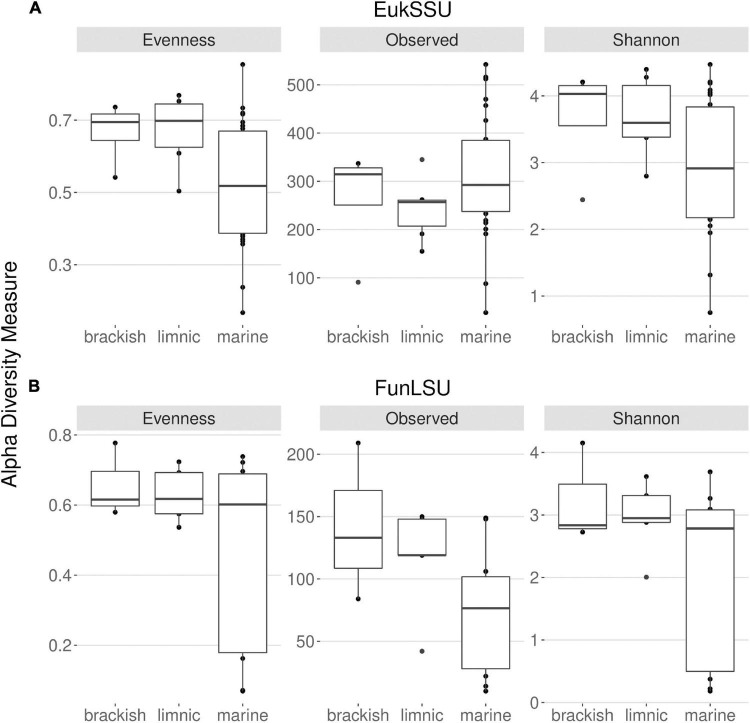
Distribution of the alpha diversity estimators according to sampled water types in **(A)** 18S and **(B)** LSU data. This includes Pielou’s evenness, observed ASV richness and Shannon diversity index.

With this study it was intended to highlight the prevalence of fungi in Antarctic benthic environments, determine the impact of glacial meltwater on benthic diatoms and their associated fungal communities and to identify correlation in their presence. To this end, freshwater, brackish and marine benthic environments were sampled and two datasets obtained: EukSSU containing the whole eukaryotic community and FunLSU containing fungal community. When examining the EukSSU community composition, a total of 3,790 sequence variants (ASVs) were identified of which 145 were classified as fungi. The relative proportion of these 145 fungal ASVs varied between different water types, from 0.97% in marine, 7.4% in brackish, to 8.9% in limnic habitats. LSU primers were not strictly fungi-specific and out of 1,204 identified ASVs, of which 727 were fungi, the remaining identified as Metazoa (262), Stramenopiles (131), Amoebozoa (35), Chlorophyta (33), Rhodophyta (12), and Alveolata (4). Before any downstream analysis, all non-fungal sequences were removed from FunLSU dataset. Nevertheless, relative proportions of fungal ASVs in FunLSU confirmed differences observed in EukSSU and 51.3% of sequences were classified as fungi in marine, 73.2% in brackish and 69.3% in limnic environments. Calculating alpha diversity measures, we note that eukaryotic and fungal diversity were lower in marine samples compared to either brackish or limnic samples (Figure 2). For eukaryotes, the dominance of a few taxa was the largest impact on diversity in marine environments, with Pielou’s evenness higher for eukaryotic communities in brackish and limnic samples (Figure 2A). In contrast, fungal communities had more even species composition amongst all three water types, with marine samples being less diverse compared to brackish or limnic samples (Figure 2B). Due to artificial variation introduced through subsampling, the omission of valid data through loss of sequence counts, or exclusion of samples with small library sizes, sequences were not rarefied prior to calculating alpha diversity (Cameron et al., 2021). As Willis (2019) discussed, environments can be identical with respect to one alpha diversity metric, but the different abundance structures will induce different biases when rarefied.

### Fungal Community Composition

**FIGURE 3:**
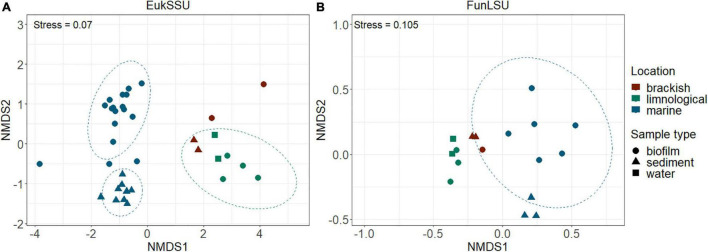
NMDS multivariate clustering of communities according to location and sample type. In both **(A)** EukSSU and **(B)** FunLSU datasets marine samples separated from limnic (*post hoc* pairwise *t*-test, *p* = 0.003) and brackish samples (*post hoc* pairwise *t*-test, *p* = 0.006) as did biofilm from sediment samples (*post hoc* pairwise *t*-test, *p* = 0.003).

To determine community composition, we carried out a non-NMDS analysis and it confirmed a distinct separation based on sample habitat (marine vs. brackish vs. limnic) (PERMANOVA, *p* = 0.001) in both EukSSU (Figure 3A) and FunLSU (Figure 3B) datasets. Marine samples were separated from limnic (*post hoc* pairwise *t*-test, *p* = 0.003) and brackish samples (*p* = 0.006), but no significant difference was observed between limnic and brackish sites. We detected a difference based on sample type (biofilm vs. sediment vs. water) (PERMANOVA, *p* = 0.001). *Post hoc* tests confirmed a difference between biofilm and sediment samples (*p* = 0.003). Summary of all the statistical tests performed can be found in Supplementary Table 1 for EukSSU dataset and Supplementary Table 2 for FunLSU dataset.

**FIGURE 4:**
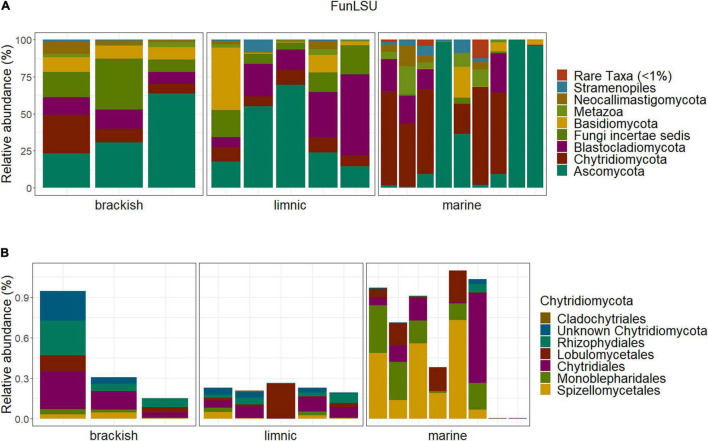
Community composition in the Antarctic benthic habitats, considering the abundance distribution in composition in sampled water types **(A)** of the whole fungal community **(B)** and the phylum Chytridiomycota relative to the total fungal community.

**FIGURE 5:**
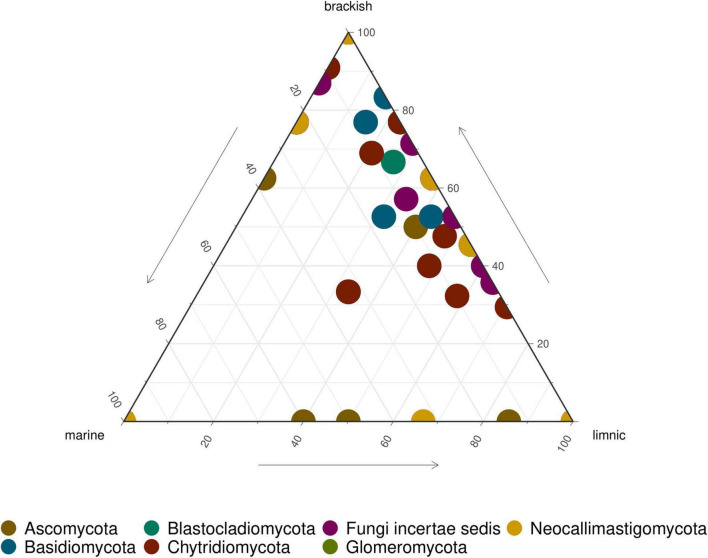
Ternary plot showing relative abundance of fungal ASVs in LSU data. Each point represents an ASV. The position represents the relative abundance of the ASV with respect to each environment.

Ascomycota and Chytridiomycota were the most abundant taxa (FunLSU dataset) in marine Antarctic benthic habitats, with no apparent distinction based on habitat or sample type (Figure 4A). Ascomycota represented 49% of the fungal reads and 25.4% of the fungal ASVs, while Chytridiomycota represented 22.4% of the fungal reads and 38.9% of the ASVs. In addition, a high diversity of the phylum Chytridiomycota was observed (Figure 4B), with Spizellomycetales and Monoblepharidales being the most abundant. In the EukSSU dataset, it was noted that fungi were more dominant in freshwater and brackish environments (8.9 and 7.4%), relative to marine environments (0.97%) (Figure 5). This was consistent with an increased diversity of fungi, as observed in both the EukSSU and FunSSU datasets.

### Co-occurrence of Fungal Parasites and Their Hosts

Using microscopy, we observed Calcofluor White stained sporangia attached to benthic algae which indicated active fungal infections (Supplementary Figure 1). Furthermore, we performed a co-occurrence network analysis to identify correlations between fungi and benthic algae to identify putative hosts. Due to the compositional nature of both the EukSSU and FunLSU datasets, it was not possible to estimate a direct linear or sparse correlation between the two datasets. Rather, we calculated sparse correlations amongst ASVs within each dataset, clustered ASVs into modules, extracted Eigen values for each module and used these values to identify correlated modules. In total, we identified seven modules in FunLSU and eight modules in EukSSU dataset. Modules largely reflected differences between habitats. FunLSU modules typically always contained at least one Chytrid ASV (Supplementary Figure 2), with Mesochytrium, Maunachytrium, Betamyces, and Spizellomyces being most abundant. For four out of the six FunLSU modules containing chytrids, we observed a correlation between that module and a EukSSU module containing a high abundance of diatoms (Supplementary Figure 3). For two FunLSU modules that contained chytrids, we did not observe a correlation with a EukSSU module, although all EukSSU modules contained diatoms (Supplementary Figure 4).

**FIGURE 6:**
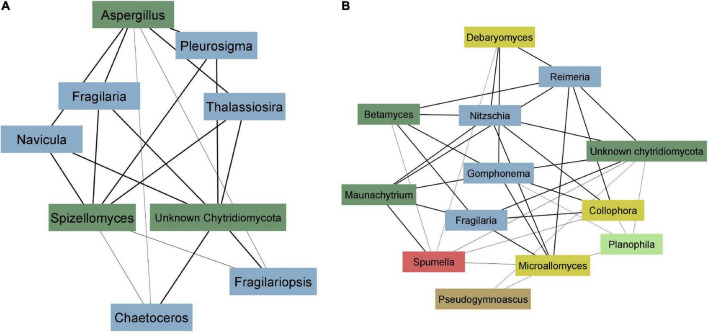
Co-occurrence network analysis. **(A)** EukSSU3 and FunLSU5 modules **(B)** EukSSU4 and FunLSU2 module. Blue—Baccilariophyceae; dark green—Chytridiomycota; yellow—other Fungi; brown—Opisthokonta, red—Stramenopiles; light green—Chlorophyta.

To better illustrate the correlation between diatoms and zoosporic fungi, we visualized weighted correlation coefficients amongst ASVs obtained from two EukSSU and FunSSU module pairs. In EukSSU3 and FunLSU5 module pair (Figure 6A) we visualized only those correlations that had a correlation value higher than 0.2. Both modules contained ASVs enriched from marine benthic environments. Specifically, we observed *Fragilariopsis cylindrus*, a pennate, benthic sea-ice diatom found both in Arctic and Antarctic waters (Helmcke and Krieger, 1953), three different *Navicula* spp. (*N. phyllepta*, *N. rhynchocephala*, *N. perminuta*) and *Pleurosigma intermedium*, all marine benthic diatoms that could serve as potential hosts for parasitic chytrids. Modules EukSSU4 and FunLSU2 (Figure 6B) contained ASVs enriched in brackish/limnic benthic habitats. In benthic environments, we observed a diverse number of benthic diatoms associated with some Chytridiomycota. In this case, we observed Betamyces (Rhizophydiales), chytrids mostly recognized as parasites on different phytoplankton species (Christaki et al., 2017), and Maunachytrium, for which there is no solid evidence for a specific lifestyle. Most abundant in both networks were ASVs classified as unknown Chytridiomycota, and they were highly correlated with certain diatom ASVs.

## Discussion

Climate warming in Antarctic environments has been associated with glacier retreat and increased ice melting (Lee et al., 2017) which, in turn, change the vertical structure of the water column, especially in Antarctic shallow coastal environments. During the summer months (December—March), meltwaters on the Western Antarctic Peninsula, where this study was undertaken, occur regularly. It has been shown that meltwater transports high particle loads to the coastal water thereby reducing incident light conditions and salinity that affect microbial community dynamics, leading to changes in species composition (Schloss et al., 2008; Antoni et al., 2020). In this study, we showed different proportions of fungal sequences between different water types. Hence, we assume a shaping impact of salinity on both the eukaryotic and fungal diversity in Potter Cove. Brackish sites, receiving strong inputs of fresh meltwater, exhibited a higher eukaryotic and fungal diversity than that of marine sites. Moreover, many of the same fungal taxa/ASVs found in freshwater environments were also determined in brackish habitats, suggesting fresh meltwater runoff, including terrestrial soil particles, might be a direct source of this increased diversity. Furthermore, it is tempting to speculate that the increased richness of fungi and the decreased dominance of individual algal hosts in brackish/freshwater environments, as shown with alpha diversity measures, might be linked. We argue that this observation can be directly linked with fungal parasites playing an active role in maintaining the diversity of algae (van Donk, 1989; Sime-Ngando and Twiss, 2012). Alternatively, this might be indirect in the sense that fertilization (Schloss et al., 2008) opens up new niches for algae, with fungal diversity, of both saprotrophic and/or parasitic fungi, promoted by an increase in the diversity of autochthonous and allochthonous organic matter (Wang et al., 2017). Finally, we conclude that the increased evenness in brackish/freshwater habitats compared to marine habitats may indicate that glacial meltwaters reduce selective pressure and increase niche opportunities for algae, possibly due to increased nutrients as observed by Death et al. (2014). In contrast, observed differences in fungal diversity may have arisen from a decreased richness in marine habitats relative to brackish/freshwater.

Our results are supported by the study of Kilias et al. (2020), who showed that chytrid fungi are primarily encountered at sites influenced by sea ice melt. The evolutionary history of Chytridiomycota suggests that they originated under brackish-/freshwater-like conditions and thus evolved successively in parallel with the host organisms or existing food (Berbee et al., 2017). It remains an open question whether marine Chytridiomycota proliferate due to lower competition in high salinity water or whether specialization of some taxa has occurred over an evolutionary time scale (Yang et al., 2021). Moreover, a substantial proportion of fungal ASVs in our FunLSU dataset were classified as terrestrial fungi. For example, Spizellomycetales, the most abundant genera among chytrids, are described as soil-inhabiting fungi (Barr, 1980). As a result, we hypothesize that spores and fungal hyphae may arrive in Antarctic coastal waters via glacial/terrestrial runoff from ice melting followed by deposition due to sedimentation alongside other particles. In particular, larger areas of bare soil at higher temperatures lead to an increased deposition of soil particles on the melting glaciers, staining them dark and even decreasing their albedo and increasing their melting (Schmitt et al., 2015). This notion is supported by the presence of Monoblepharidales, which are described as typical freshwater fungi (Sparrow, 1933), and constitute the dominant genera among fungal taxa in the sampled marine environments. Such sediment accumulation driven by increased glacier retreat may decrease the rate of microphytobenthic (MPB) primary production. As earliers discussed by Hoffmann et al. (2019), MPB might survive the consequences but their contribution to the overall primary production as a carbon resource may decline. Consequently, food competition of the benthic heterotrophic community will increase, with unpredictable consequences for biomass, density, structure and diversity of the benthic community and food web structure.

Using microscopy and performing co-occurrence network analysis, we found preliminary evidence suggesting that zoosporic fungi in Antarctic waters are involved in parasitic relationships with benthic diatoms. In summary, in most cases, parasitic chytrids were associated with the presence of Bacillariophyceae, but some diatom taxa occurred in some cases independent of parasitic chytrids. Furthermore, unclassified Chytridiomycota and Spizellomyces, which are known as terrestrial parasites on other chytrid species and soil nematodes (Lozupone and Klein, 2002), were non-specifically associated with multiple potential diatom hosts. It is tempting to speculate that these chytrids exhibit a parasitic lifestyle in aquatic habitats with broad host tolerance, considering that these ASVs represent a large hidden diversity. Whilst network approaches may provide hints for interactions between fungi and diatoms, these are ultimately correlations. Cultivation and targeted single cell approaches that identify host-parasite pairs and better discriminate between parasitic and saprotrophic interactions are still needed (van den Wyngaert et al., 2017). Despite this, our study highlights that diatom-parasite interactions are likely in polar benthic environments and may have significant ecological roles.

## Conclusion

Our work suggests that salinity has a major impact in shaping eukaryotic microbial communities in shallow coastal waters of Antarctica. In particular, we determined a significant difference in eukaryotic and fungal diversity between sampled habitats, with both eukaryotic and fungal diversity higher in brackish/freshwater environments. On the one hand, considering the potential parasitic lifestyle of dominant fungal taxa, we may conclude that fungal parasites play an active role in maintaining the diversity of benthic algae. On the other hand, increased ice melting during summer months may cause nutrient loading in brackish waters resulting in increased diversity. It remains an open question whether the positive effect on fungal diversity also has a positive or a negative effect on the ecosystem on a broad-scale, considering parasitic interactions. The factor of inputs of meltwater is also supported by the fact that freshwater and brackish environments shared many of the same fungal taxa/ASVs and that a substantial proportion of fungal ASVs in the FunLSU dataset were classified as terrestrial fungi. In terms of parasitic lifestyle, while many of the chytrid ASVs were found to be non-specifically associated with multiple potential diatom hosts, drawing mechanistic conclusion about host-parasite interactions is difficult because the most abundant ASVs in our networks were classified as unknown Chytridiomycota. Systematic analysis of fungal diversity in Antarctic regions is still in its infancy, and the taxonomy and ecological functions of Chytridiomycota species remain largely unexplored.

Our findings are a significant step toward illuminating the diversity of fungal communities and occurrence of, what it may be, fungal parasites in the Antarctic region. Benthic diatoms play a key role in polar food webs. Hence, studying chytrid diversity and their interactions with the algal hosts will provide us with a comprehensive understanding of polar ecosystems. The effects of salinity changes on fungi-microalgae interactions and microalgal communities, as well as its consequences for the coastal ecosystem of Potter Cove, are therefore subject to future studies.

## Data Availability Statement

The original contributions presented in the study are publicly available. This data can be found here: (https://www.ebi.ac.uk/ena/browser/view/PRJEB49266/PRJEB49266).

## Author Contributions

AL, H-PG, MLQ, GLC, JZ, UK, and TW organized an expedition to Antarctica and collected samples. AL performed microscopy and extracted DNA from collected samples. DI and JW analyzed the data. DI wrote the manuscript. SVdW assisted with her knowledge during the analysis. H-PG, JW, MLQ, GLC, JZ, UK, TW, and SVdW edited and provided feedback on the original draft. All authors approved the submitted version.

## Conflict of Interest

The authors declare that the research was conducted in the absence of any commercial or financial relationships that could be construed as a potential conflict of interest. The handling editor declared a past co-authorship with one of the authors UK.

## Publisher’s Note

All claims expressed in this article are solely those of the authors and do not necessarily represent those of their affiliated organizations, or those of the publisher, the editors and the reviewers. Any product that may be evaluated in this article, or claim that may be made by its manufacturer, is not guaranteed or endorsed by the publisher.

## Abbreviations

EukSSU: dataset containing whole eukaryotic community
FunLSU: dataset containing only fungal community.
