# Supplementary material for: Antarctic Glacial Meltwater Impacts the Diversity of Fungal Parasites Associated With Benthic Diatoms in Shallow Coastal Zones
Source: Front Microbiol. 2022 Mar 4;13:805694. doi: 10.3389/fmicb.2022.805694 (PMC8931407; doi:10.3389/fmicb.2022.805694)
Supplement: Supplementary file 1 [file Data_Sheet_1.docx]

Supplementary Material

# DNA extraction protocol

Zirconium and glass beads of various diameter (0.1, 0.7 and 1 mm) and 0.6 mL CTAB (cetyltrimethyl-ammonium bromide) buffer were added to ~ 0.5 g of the sample. Then 60 µl of 10% sodium dodecyl sulfate (SDS), 60 µl of 10% N-Lauroylsarcosin and 20,8 µl Proteinase K (10 mg/ml) were added. Samples were incubated for 1 h at 60 °C. After incubation 0,5 ml Phenol-Chloroform-Isoamylalkohol (25:24:1) mixture was added in each tube. Samples were then homogenized on a vortexer for 10 min at highest speed and then centrifuged at 16000×g for 10 min at 4 °C. The aqueous phase was transferred into new reaction tubes, washed with 0,6 mL of chloroform-isoamylalcohol (24:1) and centrifuged at 16000× g for 10 min at 4 °C. Again, the aqueous phase was transferred into new reaction tubes and mixed with 1200 µl of 30% polyethylene glycol (w:v) in 1.6 M NaCl and 2 µl of LPA was added. After 1 h incubation at room temperature, samples were centrifuged at 17000× g for 60 min at 4 °C. The supernatant was removed and the pellet was washed with 800 µl of ice-cold 70% ethanol. After centrifugation at 17000×g for 10 min the supernatant was removed and the nucleic acid pellet was air-dried and finally dissolved in 50 µL ultra-pure water.

# Supplementary Tables

Supplementary Table 1. Summary of statistical tests performed on EukSSU dataset, using 999 permutations and based on Bray-Curtis dissimilarity.

| PERMANOVA | | | | | | | |  |
| --- | --- | --- | --- | --- | --- | --- | --- | --- |
| Location | | | | | | | |  |
|  | Df | SS | MS | F.Model | R2 | Pr(>F) |  |  |
| Location | 2 | 2.923 | 1.46151 | 4.0462 | 0.19693 | 0.001 | *** |  |
| Residuals | 33 | 11.92 | 0.36121 | 0.80307 |  |  |  |  |
| Total | 35 | 14.843 | 1 |  |  |  |  |  |
| --- |  |  |  |  |  |  |  |  |
| Type | | | | | | |  |  |
|  | Df | SS | MS | F.Model | R2 | Pr(>F) |  |  |
| Type | 2 | 2.6205 | 1.31025 | 3.5376 | 0.17655 | 0.001 | *** |  |
| Residuals | 33 | 12.2224 | 0.37038 | 0.82345 |  |  |  |  |
| Total | 35 | 14.8429 | 1 |  |  |  |  |  |
| POST-HOC PAIRWISE T TEST | | | | | | | |  |
| Location | | | | | | | |  |
| pairs | Df | SS | F.Model | R2 | p.value | p.adjusted | sig |  |
| marine vs limnic | 1 | 1.301212 | 3.144877 | 0.094883 | 0.001 | 0.003 | * |  |
| marine vs brackish | 1 | 1.199124 | 2.938745 | 0.094986 | 0.002 | 0.006 | * |  |
| limnic vs brackish | 1 | 0.782308 | 2.085832 | 0.206808 | 0.029 | 0.087 |  |  |
| Type | | | | | | | |  |
| pairs | Df | SS | F.Model | R2 | p.value | p.adjusted | sig |  |
| biofilm vs water | 1 | 0.569538 | 1.322413 | 0.05437 | 0.137 | 0.411 |  |  |
| biofilm vs sediment | 1 | 1.556739 | 3.763204 | 0.105226 | 0.001 | 0.003 | * |  |
| water vs sediment | 1 | 0.617837 | 1.593972 | 0.126566 | 0.106 | 0.318 |  |  |
| Abbreviations: Df, degrees of freedom; SS, sum of squares; MS, mean sum of squares. Signif. Codes: 0 = ***; 0.001 = **; 0.01 = *; 0.05 = . | | | | | | | |  |
|  |  |  |  |  |  |  |  |  |
|  |  |  |  |  |  |  |  |  |

Supplementary Table 2. Summary of statistical tests performed on FunLSU dataset, using 999 permutations and based on Bray-Curtis dissimilarity.

| PERMANOVA | | | | | | | |  |
| --- | --- | --- | --- | --- | --- | --- | --- | --- |
| Location | | | | | | | |  |
|  | Df | SS | MS | F.Model | R2 | Pr(>F) |  |  |
| Location | 2 | 1.7795 | 0.88973 | 2.2078 | 0.21629 | 0.001 | *** |  |
| Residuals | 16 | 6.4478 | 0.40299 | 0.78371 |  |  |  |  |
| Total | 18 | 8.2273 | 1 |  |  |  |  |  |
| --- |  |  |  |  |  |  |  |  |
| Type | | | | | | |  |  |
|  | Df | SS | MS | F.Model | R2 | Pr(>F) |  |  |
| Type | 2 | 1.2314 | 0.61572 | 1.4082 | 0.14968 | 0.01 | ** |  |
| Residuals | 16 | 6.9959 | 0.43724 | 0.85032 |  |  |  |  |
| Total | 18 | 8.2273 | 1 |  |  |  |  |  |
| POST-HOC PAIRWISE T TEST | | | | | | | |  |
| Location | | | | | | | |  |
| pairs | Df | SS | F.Model | R2 | p.value | p.adjusted | sig |  |
| limnic vs marine | 1 | 0.851009 | 1.877151 | 0.11823 | 0.001 | 0.003 | * |  |
| limnic vs brackish | 1 | 0.642461 | 1.68885 | 0.219649 | 0.02 | 0.06 |  |  |
| marine vs brackish | 1 | 0.755645 | 1.656329 | 0.121287 | 0.006 | 0.018 | . |  |
| Type | | | | | | | |  |
| pairs | Df | SS | F.Model | R2 | p.value | p.adjusted | sig |  |
| water vs biofilm | 1 | 0.440166 | 0.911126 | 0.070569 | 0.8 | 1 |  |  |
| water vs sediment | 1 | 0.658999 | 1.590519 | 0.20954 | 0.101 | 0.303 |  |  |
| biofilm vs sediment | 1 | 0.797988 | 1.756274 | 0.111465 | 0.001 | 0.003 | * |  |
| Abbreviations: Df, degrees of freedom; SS, sum of squares; MS, mean sum of squares. Signif. Codes: 0 = ***; 0.001 = **; 0.01 = *; 0.05 = . | | | | | | | |  |
|  |  |  |  |  |  |  |  |  |
|  |  |  |  |  |  |  |  |  |

# Supplementary Figures


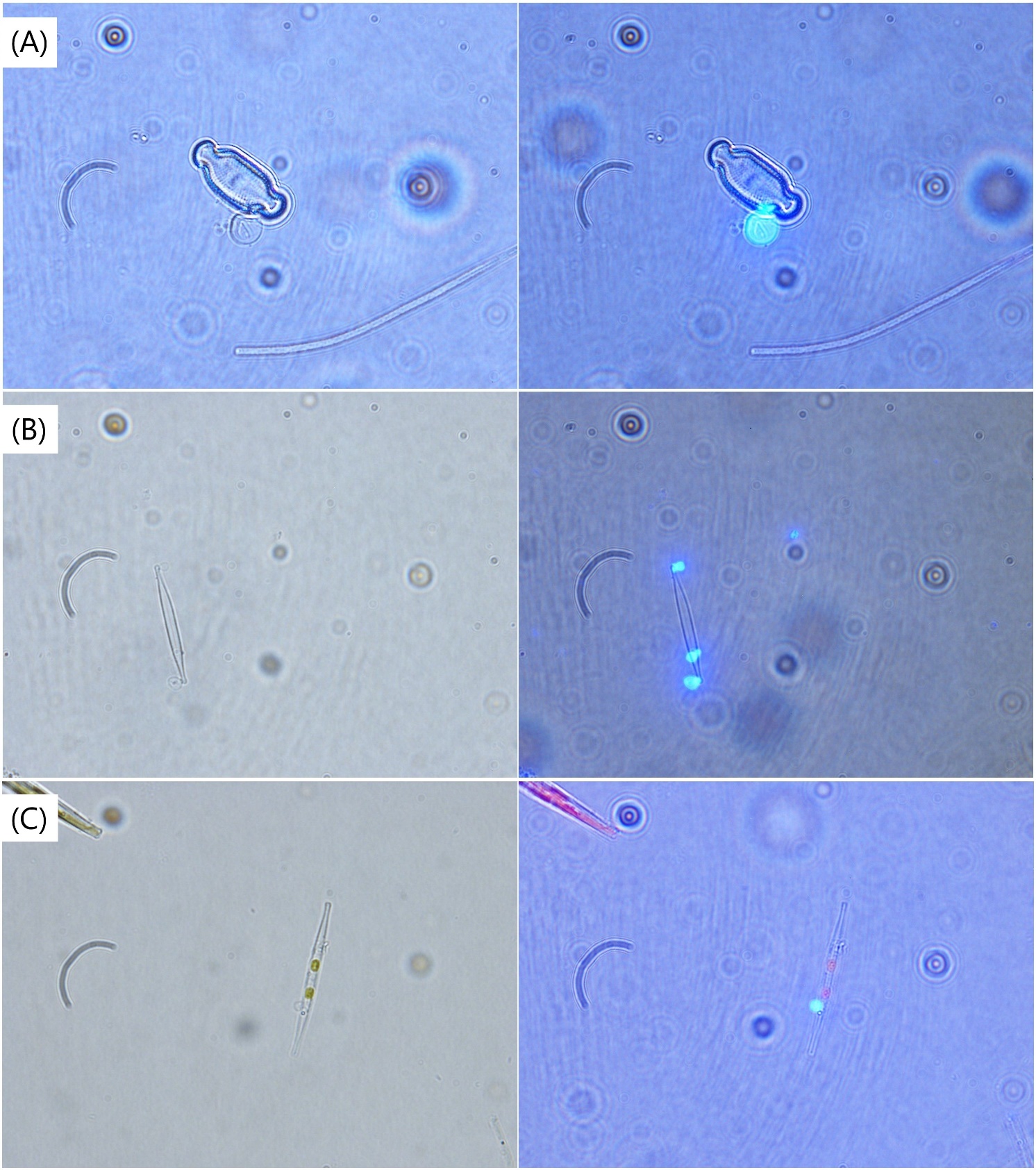


Supplementary Figure 1. (A) unidentified host cell with CFW stained sporangia (B) unidentified host cell with multiple CFW stained sporangia (C) unidentified host cell with CFW stained sporangia


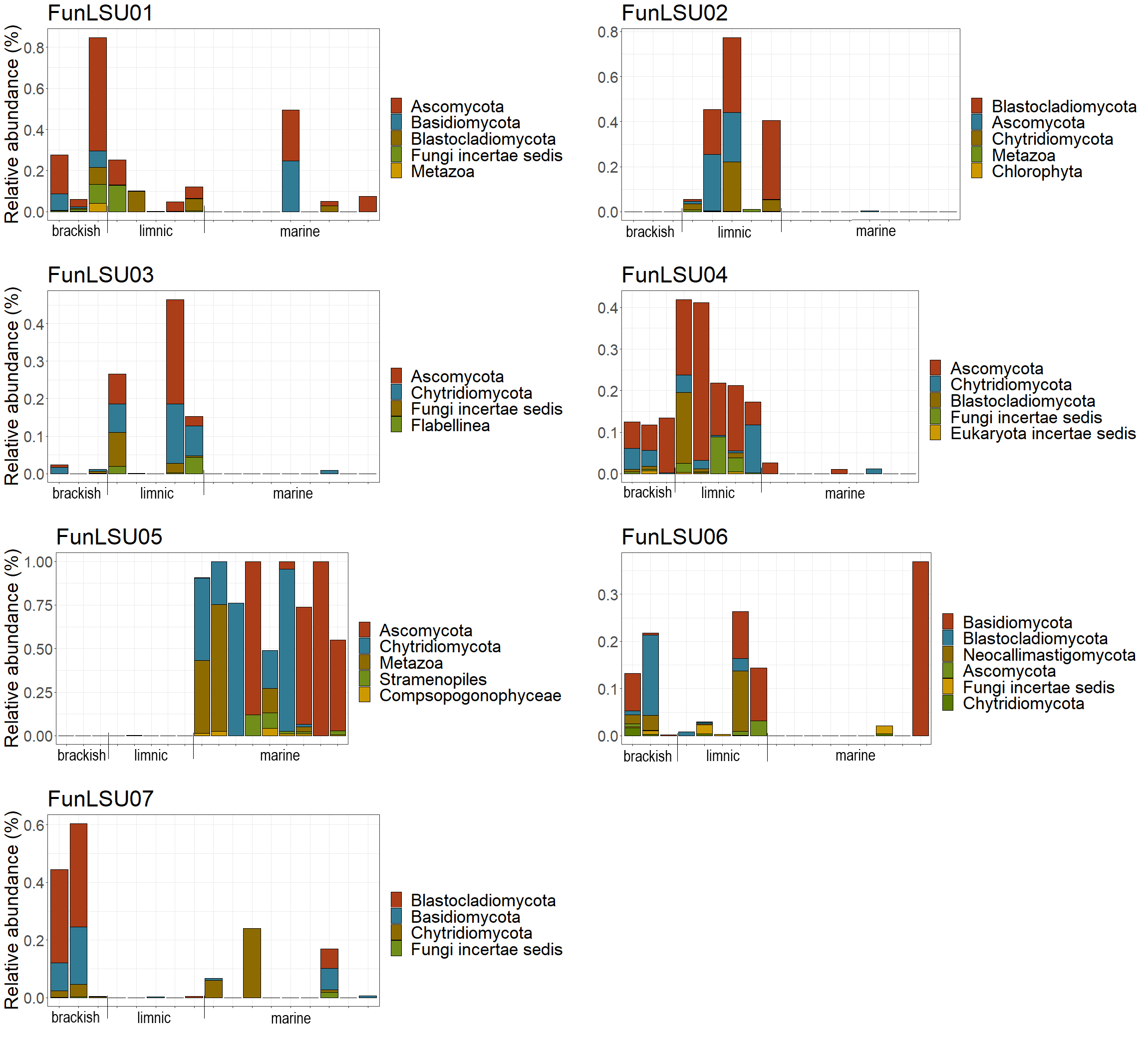


Supplementary Figure 2. Relative abundances of different groups in each module in the FunLSU dataset. Chytridiomycota were not present only in the FunLSU01 module.


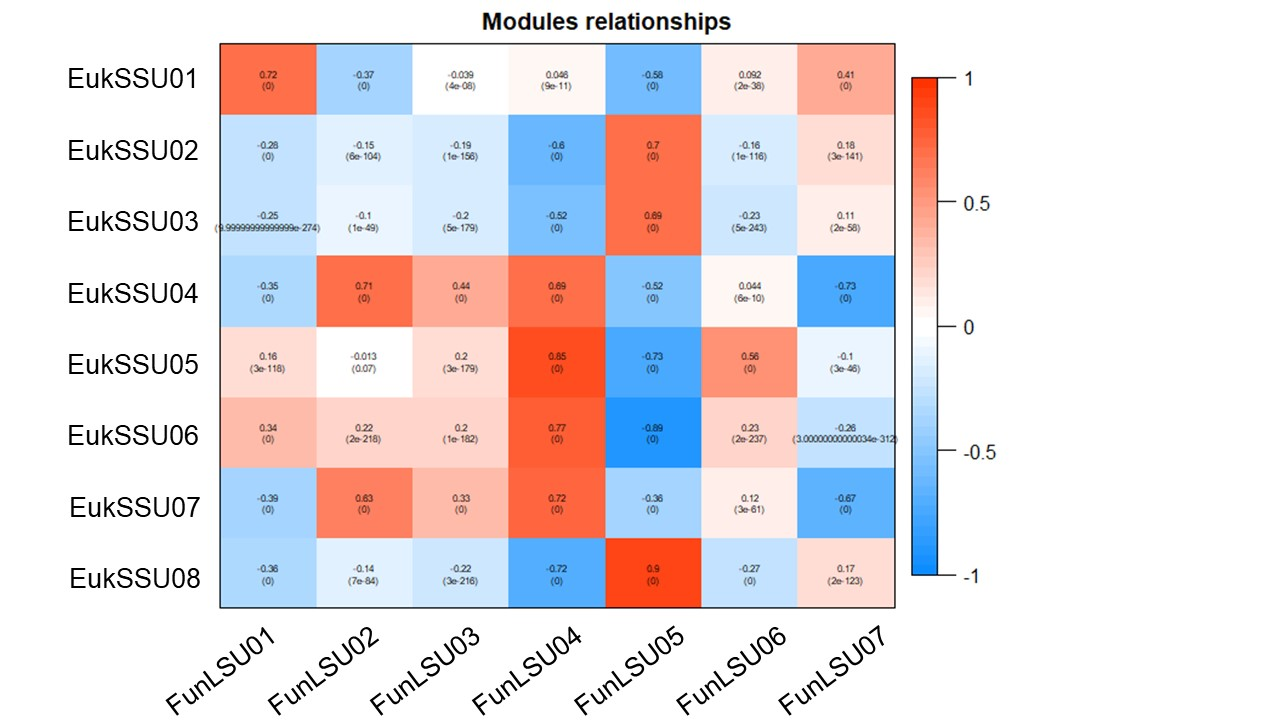


Supplementary Figure 3. Heatmap showing WGCNA EukSSU modules and their correlations to FunLSU modules. Upper numbers of each cell correspond to correlation coefficients and lower numbers in brackets to the statistical significance. Only positively correlated module pairs, with correlation coefficient > 0.5, were analyzed.


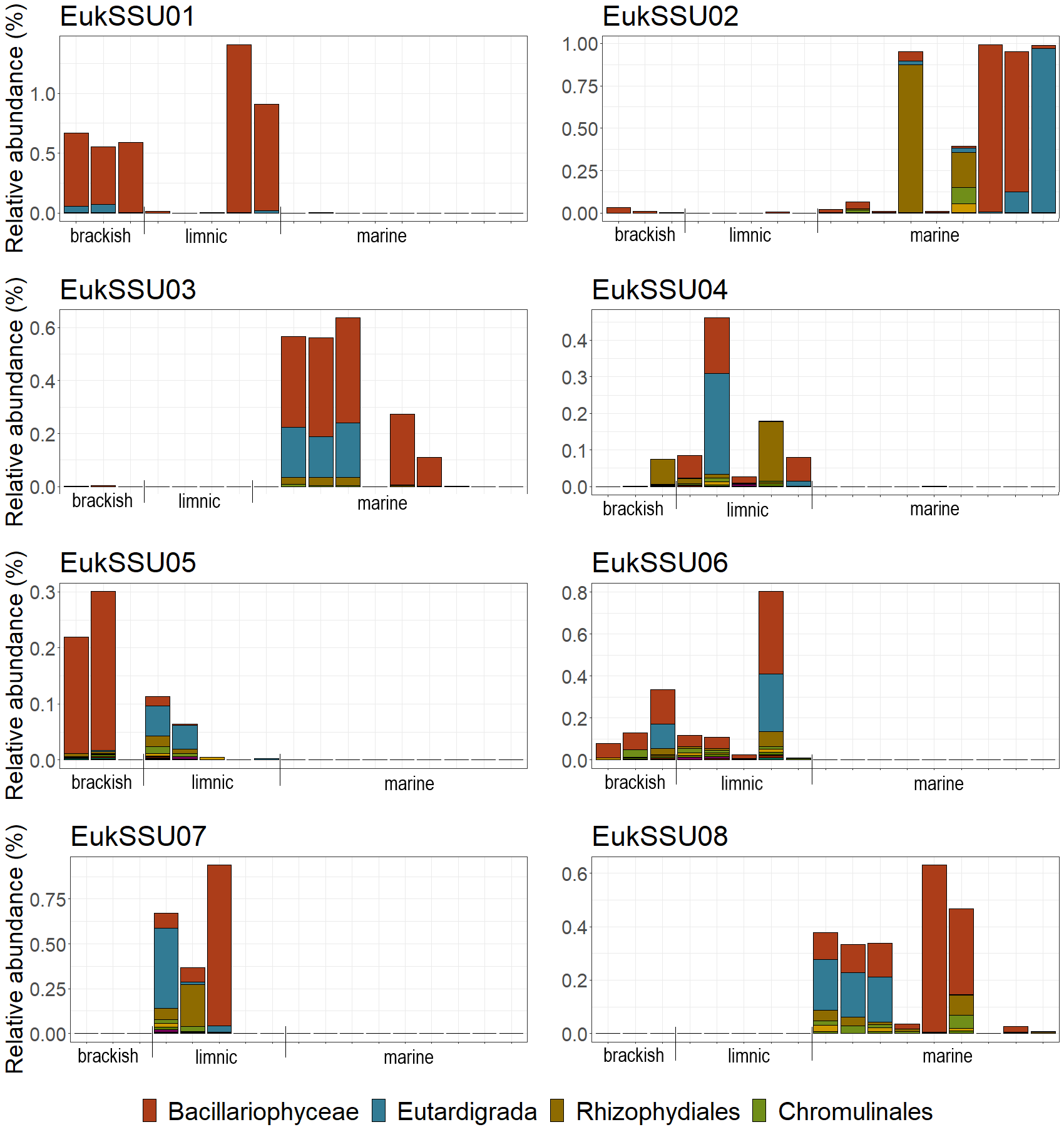


Supplementary Figure 4. Relative abundances of different eukaryotic groups common for each module in the EukSSU dataset. We focused on Bacillariophyceae as preferential hosts for fungal parasites. They were present in all EukSSU modules.
